# Performance of the Xpert MTB/RIF Ultra Assay for Determining Cause of Death by TB in Tissue Samples Obtained by Minimally Invasive Autopsies

**DOI:** 10.1016/j.chest.2020.06.071

**Published:** 2020-07-12

**Authors:** Alberto L. Garcia-Basteiro, Juan Carlos Hurtado, Paola Castillo, Fabiola Fernandes, Mireia Navarro, Lucilia Lovane, Isaac Casas, Llorenç Quintó, Dercio Jordao, Mamudo R. Ismail, Cesaltina Lorenzoni, Carla Carrilho, Ariadna Sanz, Natalia Rakislova, Aurea Mira, Miriam J. Alvarez-Martínez, Anélsio Cossa, Frank Cobelens, Inácio Mandomando, Jordi Vila, Quique Bassat, Clara Menendez, Jaume Ordi, Miguel J. Martínez

**Affiliations:** a Centro de Investigaçãoem Saúde de Manhiça (CISM), Maputo, Mozambique; b ISGlobal, Hospital Clínic, Universitat de Barcelona, Universitat de Barcelona, Barcelona, Spain; c Amsterdam Institute for Global Health and Development, Amsterdam University Medical Centers, Amsterdam, The Netherlands; d Department of Microbiology, Hospital Clinic, Universitat de Barcelona, Barcelona, Spain; e Department of Pathology, Hospital Clinic, Universitat de Barcelona, Barcelona, Spain; f Department of Pathology, Faculty of Medicine/Eduardo Mondlane University and Maputo Central Hospital, Maputo, Mozambique; g Biomedical Diagnostic Centre (CDB), Hospital Clínic, University of Barcelona, Barcelona, Spain, Instituto Nacional de Saúde (INS), Ministério da Saúde, Maputo, Moçambique; h ICREA, Catalan Institution for Research and Advanced Studies, Pg. Lluís Companys 23, Barcelona, Spain; i Pediatric Infectious Diseases Unit, Pediatrics Department, Hospital Sant Joan de Déu,University of Barcelona, Barcelona, Spain; j Consorcio de Investigación Biomédica en Red de Epidemiología y Salud Pública (CIBERESP), Madrid, Spain

To the Editor:

An estimated 1.5 million deaths were attributable to TB in 2018.^1^ However, some uncertainty exists as to the exact global figures, given that approximately 30% of incident cases are not diagnosed, and because of the difficulties of ascertaining TB as cause of death (CoD).^2^ Undoubtedly, complete diagnostic autopsies (CDAs) constitute the gold standard for establishing a diagnosis of TB at death. However, CDAs are seldom performed in high-TB-burden countries because of the scarcity of trained pathologists, the time-consuming nature of the procedure, and the meager acceptability of the practice by relatives.^3^

In recent years, an alternative minimally invasive autopsy (MIA), a procedure well accepted by the next of kin, has been developed.^4^^,^^5^ MIA can be conducted relatively rapidly with the use of biopsy needles for sampling key organs, which leave barely visible marks, which is thus more acceptable to relatives. This method has shown good sensitivity for diagnosing TB as CoD.^6^ Nonetheless, MIA has thus far used standard histological and microbiological approaches for TB diagnosis (identification of granulomatous lesions, acid-fast bacilli smears, in-house polymerase chain reaction methods),^7^ which remain time consuming, require specific expertise, and have limited sensitivity.

Thus, we evaluated the diagnostic accuracy of the molecular Xpert MTB/RIF Ultra (hereafter referred to as Xpert Ultra) assay in samples obtained by MIA to detect CoD by TB.

## Methods

This was an ancillary study to a large observational postmortem evaluation (CADMIA study) aimed at validating MIA against CDA for any CoD determination in different age groups in Maputo, Mozambique.^3^^,^^5^ Both the CDA and MIA pathological and microbiological methods have been comprehensively described elsewhere.^8^^,^^9^ In a previous analysis from CADMIA, TB-related lesions were extensively investigated in CDA samples.^6^ Microbiological methods include acid-fast bacilli smear and two molecular tools: in-house real-time polymerase chain reaction and Xpert Ultra following a pre-specified algorithm*.*^6^ CDA diagnosis was considered the gold standard for CoD determination in CADMIA.

For this specific analysis, we selected MIA samples from the lung, CNS, cerebrospinal fluid (CSF), and plasma from all the study cases with any TB finding (TB disease or *Mycobacterium TB* DNA detected in CDA samples). This analysis included a total of 117 patients. In 31 patients, TB was the final CoD, 31 cases had TB disease at death but had died of another CoD, and in 18 cases DNA of *M TB* was detected but no histological lesions compatible with TB were found. In addition, we included a subset of 37 patients with no TB findings in the CDA and with availability of the four MIA samples.

MIA samples were collected in tubes containing 1 mL lysis buffer (ATL buffer, Qiagen), and stored and processed by Xpert Ultra. Lung and CNS tissue samples were processed as described previously,^6^^,^^10^ and 0.5 mL plasma and CSF were mixed with the assay sample reagent buffer in a 1:3 ratio before testing.

We determined the performance of Xpert Ultra for each MIA sample and for combinations of samples. We also estimated the number needed to misdiagnose (NNM) as: NNM = Total/(false positives + false negatives). The NNM is the number of patients who need to be tested for one patient to be misdiagnosed. Because specific MIA samples were not available for some patients with TB findings, a sensitivity analysis of the performance of Xpert Ultra in MIA samples was conducted among cases in which all four MIA samples were available.

The Clinical Research Ethics Committee of the Hospital Clinic of Barcelona, Spain (Ref:2013/8677) and the National Bioethics Committee of Mozambique (Ref. 342/CNBS/13) approved this study.

## Results

Of the 117 cases included in this analysis, 14 patients (12.0%) were children, 85 (72.7%) were adults, and 18 (15.4%) were maternal deaths. Seventy-eight patients (67.8%) were HIV positive (HIV status could not be ascertained in two cases).

Table 1 shows the diagnostic performance of Xpert Ultra in different MIA samples and combinations of samples to diagnose TB as CoD. As a single organ, the highest sensitivity was observed in the lung (0.78; 95% CI, 0.58-0.91). The sensitivity of the test in plasma and CSF was high, being 0.68 (95% CI, 0.47-0.83) and 0.67 (95% CI, 0.30-0.67), respectively. For combinations of organs, the highest performance was observed for lung and CNS: sensitivity, 0.85% (95% CI, 0.66-0.96) and negative predictive value of 0.95 (95% CI, 0.88-0.99). The highest specificity in a single organ or fluid was obtained in the lung (0.98; 95% CI, 0.92-1.00).

**Table 1:** Diagnostic Performance of Xpert Ultra in Different Minimally Invasive Autopsy Samples to Diagnose TB as the Cause of Death

Sample/Combination of Samples	No. True Positive	No. False Negative	No. False Positive	No. True Negative	Total Samples^a^	Sensitivity	95% CI		Specificity	95% CI		PPV	95% CI		NPV	95% CI		NNM
							Lower Limit	Upper Limit		Lower Limit	Upper Limit		Lower Limit	Upper Limit		Lower Limit	Upper Limit	
Lung	21	6	2	81	110	0.78	0.58	0.91	**0.98**	**0.92**	**1.00**	**0.91**	**0.72**	**0.99**	0.93	0.86	0.97	**13.8**
CNS	21	10	4	80	115	0.68	0.49	0.83	0.95	0.88	0.99	0.84	0.64	0.95	0.89	0.81	0.95	8.2
Plasma	20	10	2	80	112	0.67	0.47	0.83	**0.98**	**0.91**	**1.00**	**0.91**	**0.71**	**0.99**	0.89	0.81	0.95	9.3
CSF	15	16	3	80	114	0.48	0.30	0.67	0.96	0.90	0.99	0.83	0.59	0.96	0.83	0.74	0.90	6.0
Lung and CNS	23	4	5	77	109	**0.85**	**0.66**	**0.96**	0.94	0.86	0.98	0.82	0.63	0.94	**0.95**	**0.88**	**0.99**	12.1
Lung and plasma	21	6	4	75	106	0.78	0.58	0.91	0.95	0.88	0.99	0.84	0.64	0.95	0.93	0.85	0.97	10.6
Lung and CSF	22	5	4	76	107	0.81	0.62	0.94	0.95	0.88	0.99	0.85	0.65	0.96	0.94	0.86	0.98	11.9
CNS and plasma	23	7	5	75	110	0.77	0.58	0.90	0.94	0.86	0.98	0.82	0.63	0.94	0.91	0.83	0.96	9.2
CNS and CSF	23	8	6	75	112	0.74	0.55	0.88	0.93	0.85	0.97	0.79	0.60	0.92	0.90	0.82	0.96	8.0
Plasma and CSF	22	8	4	75	109	0.73	0.54	0.88	0.95	0.88	0.99	0.85	0.65	0.96	0.90	0.82	0.96	9.1
Lung, CNS, & plasma	23	4	6	72	105	0.85	0.66	0.96	0.92	0.84	0.97	0.79	0.60	0.92	0.95	0.87	0.99	10.5
Lung, CNS, & CSF	23	4	6	73	106	0.85	0.66	0.96	0.92	0.84	0.97	0.79	0.60	0.92	0.95	0.87	0.99	10.6
Lung, plasma, & CSF	22	5	5	71	103	0.81	0.62	0.94	0.93	0.85	0.98	0.81	0.62	0.94	0.93	0.85	0.98	10.3
CNS, plasma, & CSF	24	6	7	70	107	0.80	0.61	0.92	0.91	0.82	0.96	0.77	0.59	0.90	0.92	0.84	0.97	8.2

The diagnostic values of samples or combinations of samples that may represent a significant diagnostic advantage are highlighted in bold. CSF = cerebrospinal fluid; NNM = number needed to misdiagnose; NPV = negative predictive value; PPV = positive predictive value.

a Some MIA samples were not tested by Xpert Ultra in some cases because of lack of remaining tissue in the biobank.

The highest NNM was obtained using the MIA sample of the lung (13.8) and combinations including lung samples (lung and CNS, 12.1; lung and CSF, 11.9; lung and plasma, 10.6). The sensitivity analysis of only cases in which the four MIA samples were available showed similar results, although the point estimate with the highest specificity (0.99; 95% CI, 0.93-1.00) and positive predictive value (PPV) (0.94; 95% CI, 0.70-1:00) was obtained with the CSF sample (Table 2).

**Table 2:** Diagnostic Performance of Xpert Ultra in Different Minimally Invasive Autopsy Samples to Diagnose TB as the Cause of Death (Includes Only Cases in Which the Four Samples Were Available: Lung, CNS, Plasma, and CSF)

Sample/Combination of Samples	No. True Positive	No. False Negative	No. Fase Positive	No. True Negative	Total Samples	Sensitivity	95% CI		Specificity	95% CI		PPV	95% CI		NPV	95% CI		NNM
							Lower Limit	Upper Limit		Lower Limit	Upper Limit		Lower Limit	Upper Limit		Lower Limit	Upper Limit	
Lung	21	6	2	73	102	0.78	0.58	0.91	0.97	0.82	0.99	0.91	0.72	0.99	0.92	0.84	0.97	**12.8**
CNS	20	7	3	72	102	0.74	0.54	0.89	0.96	0.89	0.99	0.87	0.66	0.97	0.91	0.83	0.96	10.2
Plasma	19	8	2	73	102	0.70	0.50	0.86	0.97	0.91	1.00	0.90	0.70	0.99	0.90	0.81	0.96	10.2
CSF	15	12	1	74	102	0.56	0.35	0.75	**0.99**	**0.93**	**1.00**	**0.94**	**0.70**	**1.00**	0.86	0.77	0.93	7.8
Lung and CNS	23	4	4	71	102	**0.85**	**0.66**	**0.96**	0.95	0.87	0.99	0.85	0.66	0.96	**0.95**	**0.87**	**0.99**	**12.8**
Lung and Plasma	21	6	4	71	102	0.78	0.58	0.91	0.95	0.87	0.99	0.84	0.64	0.95	0.92	0.84	0.97	10.2
Lung and CSF	22	5	3	72	102	0.81	0.62	0.93	0.96	0.89	0.99	0.88	0.69	0.97	0.94	0.85	0.98	**12.8**
CNS and Plasma	22	5	5	70	102	0.81	0.62	0.93	0.93	0.85	0.98	0.81	0.62	0.93	0.93	0.85	0.98	10.2
CNS and CSF	22	5	4	71	102	0.81	0.62	0.93	0.95	0.87	0.99	0.85	0.65	0.96	0.93	0.85	0.98	11.3
Plasma and CSF	21	6	3	72	102	0.78	0.58	0.91	0.96	0.89	0.99	0.88	0.68	0.97	0.92	0.84	0.97	11.3
Lung CNS Plasma	23	4	6	69	102	0.85	0.66	0.96	0.92	0.83	0.97	0.79	0.60	0.92	0.95	0.87	0.98	10.2
Lung CNS CSF	23	4	5	70	102	0.85	0.66	0.96	0.93	0.85	0.98	0.82	0.63	0.94	0.95	0.87	0.99	11.3
Lung Plasma CSF	22	5	5	70	102	0.81	0.62	0.93	0.93	0.85	0.98	0.81	0.62	0.93	0.93	0.85	0.98	10.2
CNS Plasma CSF	23	4	6	69	102	0.85	0.62	0.93	0.92	0.83	0.97	0.79	0.60	0.92	0.95	0.87	0.98	10.2
Any tissue	23	4	7	68	102	0.85	0.66	0.96	0.91	0.82	0.96	0.77	0.58	0.90	0.94	0.86	0.98	9.3

The diagnostic values of samples or combinations of samples that may represent a significant diagnostic advantage are highlighted in bold. See Table 1 legend for expansion of abbreviations.

## Discussion

This analysis shows that Xpert Ultra (a simple, rapid, and highly sensitive molecular tool) can be directly applied to specific MIA samples and achieve a reasonably high accuracy for confirming or ruling out TB as the CoD. We observed that only 15% of TB deaths would have been missed with the use of Xpert Ultra in lung and CNS MIA samples. More than two thirds of TB cases would have been diagnosed by performing Xpert Ultra in an easily accessible and homogeneous sample such as plasma, a sample with great potential for confirming TB as CoD given its high associated PPV (0.90 in this setting) and which would seldom be positive if the patient had not died of TB (specificity, 0.98). A similar high specificity and PPV are obtained with CSF, probably reflecting that most TB deaths are caused by disseminated TB, and bacilli are released and found in great quantities in peripheral blood and other compartments, such as CSF.

The study includes a well-characterized sample of patients with different TB findings at death. It also includes cases from different age groups and HIV statuses. Nonetheless, it has certain limitations. First, the sample size of cases with TB as the CoD was limited. Second, we could not analyze all of the CADMIA cases in this analysis because MIA samples were not available in all of the cases. Thus, the prevalence of TB as the CoD is not real in this sample, a factor that might affect the interpretation of PPV and negative predictive value. Taking into account that we included all cases with any type of TB finding in the CDA but only a subset of cases without any finding, the specificity of the MIAs might be slightly underestimated.

In conclusion, this study shows that the use of Xpert Ultra in body fluids, such as plasma or CSF, obtained at MIA, can easily and quickly diagnose or rule out TB as the CoD. This diagnostic strategy can accelerate and adequately and accurately determine CoD in settings with high TB and HIV prevalence. Nonetheless, the implications for pre-mortem patient management still need to be elucidated.
